# Recent advances in SHH medulloblastoma progression: tumor suppressor mechanisms and the tumor microenvironment

**DOI:** 10.12688/f1000research.20013.1

**Published:** 2019-10-29

**Authors:** Lukas Tamayo-Orrego, Frédéric Charron

**Affiliations:** 1 Montreal Clinical Research Institute (IRCM), Montreal, Quebec, Canada; 2 Integrated Program in Neuroscience, McGill University, Montreal, Quebec, Canada; 3 Grupo Neuroaprendizaje, Autonomous University of Manizales, Manizales, Colombia; 4 Department of Anatomy and Cell Biology, Division of Experimental Medicine, McGill University, Quebec, Canada; 5 Department of Medicine, University of Montreal, Montreal, Quebec, Canada

**Keywords:** Medulloblastoma, Sonic hedgehog, cell senescence, tumor microenvironment, tumor progression

## Abstract

Medulloblastoma, the most common of the malignant pediatric brain tumors, is a group of four molecularly and clinically distinct cancers with different cells of origin. One of these medulloblastoma groups displays activation of Sonic hedgehog (SHH) signaling and originates from granule cell precursors of the developing cerebellum. Ongoing basic and clinical research efforts are tailored to discover targeted and safer therapies, which rely on the identification of the basic mechanisms regulating tumor initiation, progression, and metastasis. In SHH medulloblastoma, the mechanisms regulating neural progenitor transformation and progression to advanced tumors have been studied in some detail. The present review discusses recent advances on medulloblastoma progression derived from studies using mouse models of SHH medulloblastoma. We focus on mechanisms that regulate progression from precancerous lesions to medulloblastoma, describing novel roles played by tumor suppressor mechanisms and the tumor microenvironment.

## Introduction

### The multi-stage nature of cancer

Cancer is a multi-step process where the development of high-grade tumors normally requires the occurrence of multiple and sequential genetic events ^1^, a process called multi-stage tumorigenesis ^2^. The paradigms of this model are adult epithelial cancers, such as pancreatic and colorectal ^3^. Each histopathological stage of the disease is followed by a more ‘malignant’ form, and the transitions are governed by a genetic event or ‘hit’ that leads to the clonal expansion of the mutated cell; within this framework, each stage can be seen as a rate-limiting step ^3^. In the case of adult epithelial cancers, an average of five to seven ‘hits’ seems to be required for the formation of a full-blown tumor ^4, 5^. It is useful to introduce here the terms ‘gatekeeper’ and ‘caretaker’ mutation to distinguish, respectively, mutations that are able to *initiate* the process of tumorigenesis and those that *promote* it or allow *progression* from one stage to the next. For example, a germ-line deletion of the *APC* gene leads to the *initiation* of colorectal carcinogenesis, and therefore *APC* is a gatekeeper for the colon epithelium. On the other hand, although *TP53* mutations appear at high frequency during colon tumorigenesis, they more rarely lead to colon carcinoma when present in the germ-line; in this context, *TP53* can be considered as a caretaker gene. Gatekeepers tend to be tissue specific, as their gene products can only deregulate the growth of the specific cell populations where they play a physiological role. For example, *VHL* is a gatekeeper in renal epithelial cells, *NF1* in the peripheral nervous system, and *MEN1* in endocrine cells ^6^. In the present work, we will see how the hedgehog receptor *PTCH1* functions as gatekeeper in neuronal precursors of the cerebellum.

### Medulloblastomas are molecularly and clinically diverse

There has been a revolution in cancer genomics in the last two decades, and neuro-oncology is not an exception. While many different pediatric brain tumors were initially aggregated together under the denomination of ‘primitive neuroectodermal tumors’, new research methods enable the classification and grouping of brain tumors and the characterization of new rare diseases ^7^. Medulloblastoma (MB) is one of the most common pediatric brain tumors and the most common malignant pediatric brain tumor. A tumor of the posterior fossa affecting mostly infants and children, MB is also diagnosed in adults ^8^. The current knowledge based on gene expression analysis allows the identification of four molecular groups of MB ^9^. These groups differ not only in terms of gene expression but also in their methylation patterns, mutational events, and clinical characteristics, such as prognosis or risk of metastasis ^8, 10– 14^. The four MB groups are called WNT-MB, Sonic hedgehog (SHH)-MB, group 3 MB, and group 4 MB. As the name indicates, WNT-MB and SHH-MB display deregulation in WNT signaling and SHH signaling, respectively. Group 3 MB has a photoreceptor/retinal expression signature, while group 4 MB expresses neuronal genes ^12^. The current idea is that each different MB group is derived from a specific cell of origin ^15, 16^, which therefore determines the clinical and molecular behavior of the disease. Additionally, the four MB groups have recently been subdivided into 12 subtypes which display specific molecular and clinical characteristics ^17^. This section discusses the mechanisms of MB formation in SHH-MB.

### Shh signaling induces granule cell precursor proliferation

**Figure 1.**
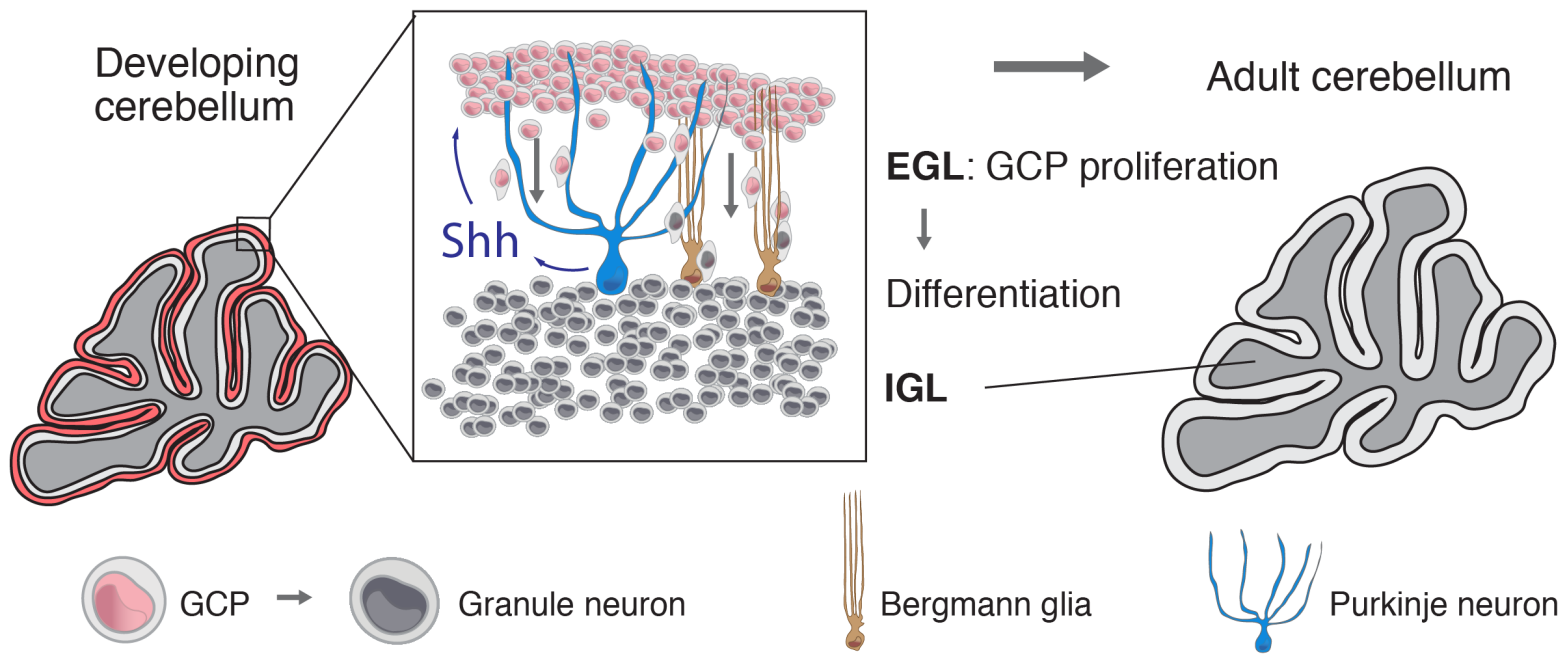
Cerebellum development. Granule cell precursors (GCPs), the progenitors that give origin to granule neurons, proliferate in the external granule cell layer (EGL) of the cerebellum in response to Purkinje neuron-derived Shh. Granule neurons populate the internal granule cell layer after GCPs differentiate and migrate through the Purkinje cell layer using the radial processes of Bergmann glia. The peak of GCP proliferation spans the first seven postnatal days in the mouse; in humans, it extends from the second half of gestation to the sixth postnatal month ^36^. IGL, internal granule cell layer.

Cerebellar granule neurons are the largest neuronal population of the nervous system ^18^. These cells arise from the granule cell precursors (GCPs), a population of cells derived from the rhombic lip that populates the surface of the cerebellum and forms the external granule cell layer (EGL) ^19^. In mice, after one week of proliferation, GCPs start to differentiate and migrate towards the internal granule cell layer (IGL) using Bergmann glia as a scaffold. The EGL disappears completely at the third postnatal week ( Figure 1) ^20^. Although different molecules promote the proliferation of neuronal precursors, Shh is the most important mitogen for GCPs ^21– 23^.

The essential components of the Hedgehog (Hh) pathway are the membrane proteins Patched1 (Ptch1) and Smoothened (Smo) as well as Gli transcription factors ^24^. Smo and Gli are activators of the pathway, while Ptch1 represses Smo activity. Shh binds to its receptor Ptch1, an event that relieves Smo repression by Ptch1 and allows the activation of Gli transcription factors ^25^. This signaling mechanism relying on the Ptch1–Smo–Gli axis is known as the canonical Hh signaling pathway. Shh-induced GCP proliferation is dependent on N-Myc transcription downstream of Gli ^26^ and the subsequent induction of D-type cyclins, which promote cell cycle progression and cell proliferation ^27, 28^.

### Mutations in Shh signaling pathway genes cause medulloblastoma

The SHH-MB group comprises 30% of all MBs ^12^. Mutations in *PTCH1*, *SMO*, and *SUFU* or amplification of *GLI1*, *GLI2*, and *N-MYC* ^8^ cause SHH-MB. *PTCH1* mutations are the most common MB driver ^29^. Mice heterozygous for *Ptch1 (Ptch1 ^+/–^* mice), or mice bearing an active form of *Smo*, develop MBs that recapitulate the human disease, demonstrating that mutations in components of the Shh pathway are responsible for MB tumorigenesis ^30, 31^. Consistent with their responsiveness to Shh signaling, GCPs have been shown through several lines of evidence to be the cell of origin of Shh-MB in mice ^32– 34^. For example, activation of Smo or deletion of *Ptch1* using the GCP-specific driver Atoh1 (also known as Math1) causes Shh-MB. MB can also be generated by Shh pathway activation in earlier cell progenitors (neural stem cells), such as GFAP- and Olig2-positive cells, but only after stem cells commit to a GCP lineage ^33, 34^. Recent single-cell RNA-seq experiments show that among all the cerebellar cell types, GCP gene expression most closely matches SHH-MB gene expression ^16^, supporting the conclusion that GCPs are also the cell of origin of SHH-MB in humans.

In rare cases, patients with Li-Fraumeni syndrome (germline *TP53* mutations) develop MB. These tumors are always SHH-MBs that display additional mutations leading to constitutive Hh pathway signaling ^35^; this requirement of Hh pathway hyperactivation for MB formation supports the idea that Hh signaling genes initiate tumorigenesis in GCPs.

## Hh signaling activity alone is not sufficient for the formation of advanced MB

In the case of activating *Smo* mutations, MB seems to develop very early from progenitors directly without transiting through a preneoplastic stage ^31^. In contrast, the removal of one *Ptch1* allele leads to the formation of precancerous lesions, an intermediate histopathological stage ^32^. In the specific case of the *Ptch1* model, it was initially thought that in MBs caused by *Ptch1* mutations, tumor formation is a two-step process where *Ptch1* haploinsufficiency causes preneoplasia and *Ptch1* loss of heterozygosity (LOH) causes constitutive Hh pathway activation, leading to MB progression ^37, 38^. Together with the facts that pediatric tumors have been considered to display a paucity of mutations, and that Smo mutations seem to cause MB in one step, many early reports suggested that Hh pathway activation (through Smo activation or complete *Ptch1* loss) may be sufficient to drive MB formation.

However, several lines of reasoning suggested that the ‘two-hit’ model of MB formation might be incorrect. If *Ptch1* haploinsufficiency leads to preneoplasia formation, why doesn’t the whole EGL of the cerebellum become preneoplastic? By definition, a preneoplastic lesion is a discrete clonal expansion and, therefore, the cells contained within it must carry an additional genetic or epigenetic event responsible for its growth (an event not present in all other *Ptch1 ^+/–^* GCPs that differentiated normally into granule neurons). Similarly, while most *Ptch1 ^+/–^* mice develop various discrete preneoplastic lesions ^38^, not all of them form an advanced MB. In fact, the incidence of preneoplastic lesions is highest at P14 and decreases progressively during subsequent weeks until approaching the incidence of advanced MB ^38^. Indeed, a longitudinal MRI study of precancerous lesions resulting from *Ptch1* deletion demonstrated that some preneoplastic lesions regress and do not become MB ^39^. In line with this reasoning, we predicted that 1) preneoplastic lesions in *Ptch1 ^+/–^* mice are not Ptch1 haploinsufficient, 2) unidentified tumor-suppressive mechanisms operate at the preneoplastic stage and limit their progression to advanced MB, and 3) additional genetic or epigenetic events regulate the progression from precancerous lesions to MB.

### Cell senescence is a tumor-suppressive mechanism in SHH-MB

In addition to the two hits discussed above ( *Ptch1* heterozygosity and *Ptch1* LOH), we identified additional events (‘third hits’) involved in MB formation: spontaneous *p53* mutations or *Cdkn2a* inactivation. We showed that preneoplastic lesions are the result of *Ptch1* LOH and not *Ptch1* haploinsufficiency ^40^. Moreover, we showed that preneoplastic lesions become senescent, therefore identifying a novel tumor suppressor mechanism for MB operating at the preneoplastic stage. Altogether, this work shows that the previous two-hit models were incomplete, since they overlooked essential components in the process of MB formation. In other words, these results demonstrate that MB molecular evolution is more complex than was previously imagined ^41^. Additional factors support a role for cell senescence or other cooperating mechanisms in MB progression. For example, the Cdk inhibitor p27 ^Kip1^ regulates the rate of MB formation in SmoA1 mice without affecting tumor incidence ^42, 43^, indicating that p27 ^Kip1^ is an additional regulator of MB progression. In some cases, senescence responses in MB result from cell stress. Citron kinase inactivation has been shown to induce cytokinesis failure, cell death, and cell senescence in SmoA1 MB, as demonstrated by increased p27 ^Kip1^, p21, and p16 ^ink4a^ *(Cdkn2a)* levels ^44^. ATR deletion also reduces MB formation upon constitutive Smo activation ^45^.

The fact that other events and mechanisms are necessary for MB formation in addition to Hh pathway activation can change the way new MB therapies are imagined and orient research in new directions. For example, most early efforts in designing therapies were aimed at inhibiting SMO or GLI transcription factors ^46, 47^. The fact that new mechanisms are at play means that additional tumor mechanisms could be targeted or exploited in order to treat MB or prevent its progression.

### SHH-MB age of onset and cell senescence

Different triggers of cell senescence exist. While oncogene-induced cell senescence (OIS) is a form of senescence induced by oncogenic stress that requires and is mediated by activation of the DNA damage response (DDR) ^48^, proliferative or oncogenic signals can also induce telomere attrition and replicative senescence (RS) ^49^. This implies that different types of senescence require different mechanisms of senescence bypass. It is commonly accepted that mutations in DDR genes such as *TP53* or *ATM* evade OIS ^50^, while telomerase ( *TERT* promoter) mutations restore telomere function and bypass RS ^51^.

**Figure 2.**
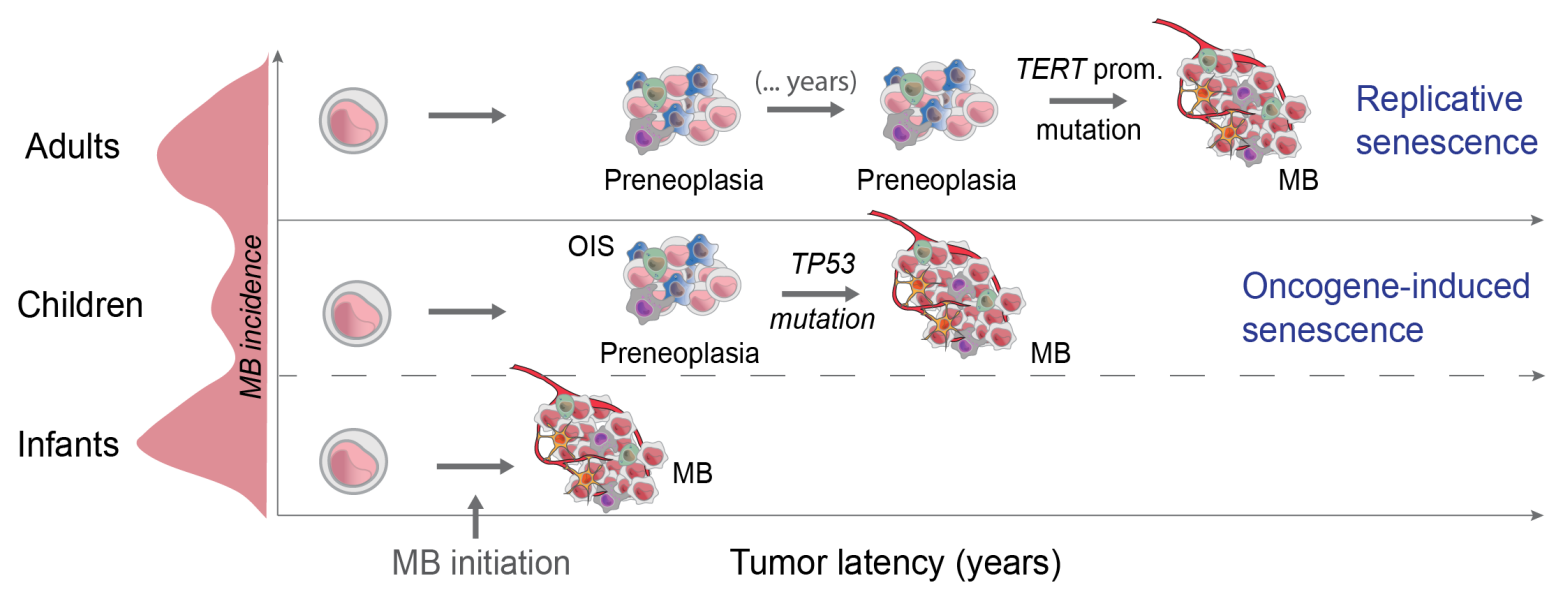
Different cell senescence mechanisms could explain tumor latency differences in SHH medulloblastomas (SHH-MBs). While *TP53* mutations are enriched in child SHH-MB, *TERT* promoter mutations exclusively affect SHH-MB diagnosed in adults. We speculate that different cell senescence mechanisms underlie differences in MB latency. One possibility is that slow-growing precancerous lesions undergo replicative senescence (RS) that needs to be evaded by *TERT* promoter mutations in adult cases. In children, oncogene activation causes fast proliferation and oncogene-induced senescence (OIS), driving the acquisition of *TP53* mutations or *p16 ^ink4^* inactivation.

In terms of the mutation landscape, adult SHH-MBs are different compared to infant and child SHH-MBs ^52^. Virtually all adult SHH-MBs with *PTCH1* mutations display *TERT* promoter mutations, while they are absent from infant/child SHH-MBs with *PTCH1* mutations ^29^. This strongly suggests that adult SHH-MBs undergo RS, consistent with the idea that those tumors develop over a longer period of time ( Figure 2); this idea is also supported by the higher mutational load of adult SHH-MBs ^52, 53^, indicative of longer tumor latency. In contrast, SHH-MBs in children are enriched in *TP53* mutations ^17^, consistent with the idea that SHH-MBs in younger patients undergo OIS.

**Figure 3.**
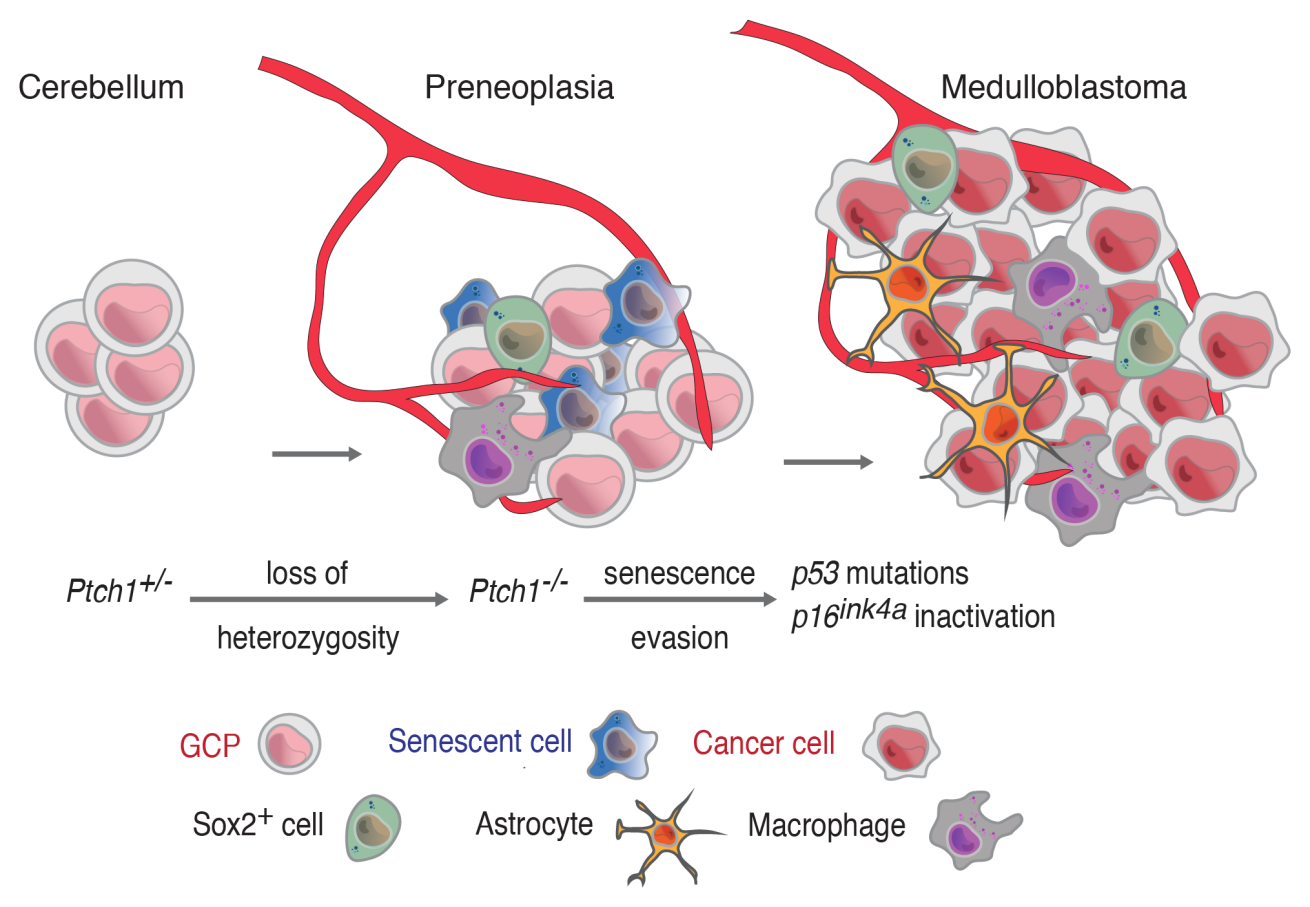
Medulloblastoma formation in *Ptch1 ^+/–^* mice and components of the tumor microenvironment Besides cancer cells, preneoplastic lesions and advanced medulloblastoma contain multiple cell types that form the tumor stroma. Preneoplasia contains senescent cells and recruits the formation of blood vessels and immune cells, including macrophages. The cell types composing this tumor microenvironment regulate preneoplasia progression to advanced tumors. GCP, granule cell precursor.

The existence of cell senescence in MB preneoplasia has potential implications and opens new avenues of research. Although in our experiments cell senescence operates as a tumor-suppressive mechanism, the functions and potential roles of senescent cells in SHH-MB are presently unknown. Whether preneoplastic senescent cells contribute or respond to environmental changes in other preneoplastic cells is not known; additionally, whether senescent and resident stem-like cells interact remains an open question. These questions are discussed below ( Figure 3).

## Emerging players in MB progression

### Tumor microenvironment

Recent work has investigated the role of the tumor microenvironment in MB progression. Brain tumors are composed of not only tumor and stem-like cells but also tumor-associated components (stroma) such as vascular and immune cells, astrocytes, microglia, and extracellular matrix ^54^. This has potential clinical implications, since tumor-associated cells could be the focus of targeted therapies.

One of the key features distinguishing SHH-MB among other MB groups is its frequent desmoplastic histology ^8^. Desmoplasia is defined as the growth of fibrous or connective tissues and therefore reflects the presence of tumor stroma. Surprisingly, little is known about the causes of this tumor histological phenotype in SHH-MB. However, some interesting insights can be drawn from the study of other cancer types in which Shh signaling induces desmoplasia, such as prostate and pancreatic cancers ^55^. In these epithelial cancers, tumor cells produce Shh, which signals to the stroma and promotes tumor growth ^56, 57^. These studies highlighted the crosstalk mediated by Shh between cancer cells and other cell components within tumors.

A recent report studied gene expression signatures and designed algorithms to extrapolate the relative contribution of cells from the tumor microenvironment in groups of MB and relative to other brain tumor entities. This study found that MBs display low expression of immune markers (including PD1, a cell-surface immune checkpoint receptor) compared to other brain tumors ^58^. Additionally, it was found that SHH-MBs display signatures predictive of fibroblast, T cell, and macrophage infiltration ^58^, findings corroborated by flow cytometry studies ^59^. Interestingly, studies are starting to show functional roles for these cell types in MB tumorigenesis and are summarized in this section.

Macrophages are the most abundant type of immune cells in brain tumors ^54^. Among the MB groups, SHH-MB displays the highest number of macrophages ^60^. Tumor-associated macrophages (TAMs) have been shown to promote or restrain tumor growth depending on the context. In astrocytomas, TAMs have been shown to release cytokines that facilitate tumor cell proliferation and survival and therefore display tumor-promoting roles ^61^. Two kinds of macrophages, M1 and M2, have traditionally been described. A first report indicated that human SHH-MBs displaying high numbers of M1 macrophages display worse prognosis ^62^. However, a recent study in mice using genetic and cell biology approaches demonstrated that TAMs in Shh-MB caused by SmoA1 activation display tumor cell killing properties and therefore have a tumor suppressive role ^63^; TAMs in Shh-MB corresponded predominantly to bone marrow-derived monocytes that invaded the MB tissue during tumor growth ^63^. Additional studies will be required to determine specific functional roles of macrophage subtypes in MB as well as the relative contribution of microglia to tumor formation.

T lymphocytes also infiltrate primary MB ^64^. In orthotopic transplantation experiments, it was shown that MB cells trigger the secretion of the T cell chemoattractant RANTES from the endothelium, leading to T cell migration ^64^. Other studies using transgenic mouse models have shown that Shh-MB tumor cells produce TGF-β, which leads to reduced CD8 T lymphocyte expansion and activation, limiting anti-tumor cytotoxic activity ^65^. MB-bearing mice also display low numbers of peripheral T cells and smaller spleens, indicating that MB affects immune function systemically ^65^. Consistent with these findings, high numbers of T helper lymphocytes (TH1) as well as high levels of IFNγ and TNFα correlate with good prognosis in human MB patients undergoing chemotherapy and hematopoietic stem cell transplantation ^66^. While these findings suggest that MB cells modulate and perhaps inhibit T cell-dependent responses, further work is required to determine the specific functions of T cell subpopulations and NK (natural killer) cells in MB pathogenesis and whether they could be exploited for therapies.

Astrocytes are abundant in human SHH-MBs and in various mouse models of Shh-MB. Tumor astrocyte-derived Shh was shown to induce the proliferation of advanced MB tumor cells from *Atoh1-Cre*; *Ptch1 ^fl/fl^* mice, preventing their differentiation and leading to increased tumor growth ^67^. In this case, it seems that tumor cells exploit the normal Shh-secreting function of astrocytes to their advantage. It is unknown whether other astrocyte functions, such as maintaining tissue homeostasis, also play a role in MB tumorigenesis. Additionally, whether tumor-associated astrocytes are recruited from other central nervous system (CNS) regions and whether they are related to Bergmann glia or are perhaps derived from MB stem cells remain to be determined.

### Role of the vasculature and extracellular matrix

The presence of the blood brain barrier (BBB) distinguishes the vasculature of the CNS. Composed of endothelial cells, pericytes, and astrocyte processes, the BBB isolates the brain parenchyma from toxic circulating compounds, including chemotherapeutics ^54^. In contrast to WNT-MB, SHH-MB displays a BBB, which seems to be induced by tumor cells ^68^. This process is controlled by Wnt signaling in vascular endothelial cells. Importantly, it has been shown that Norrin/Frizzled signaling regulates angiogenic remodeling and acts as a tumor-suppressive mechanism; in the absence of Fzd4 signaling, angiogenic changes led to increased cell proliferation, reduced apoptosis, and increased rates of *Ptch1* LOH and progression to advanced tumors in *Ptch1 ^+/–^* mice ^69^.

*In vitro* studies of endothelial cells show that Shh promotes blood vessel formation via cytoskeletal remodeling ^70^. In the healthy brain, Hh signaling promotes BBB formation and integrity: astrocyte-derived Shh signals to endothelial cells, where it decreases trans-endothelial permeability and promotes immune quiescence by decreasing inflammatory mediators and leukocyte migration ^71^. This role of Shh perhaps explains the relatively low levels of inflammatory markers in SHH-MB compared to other molecular MB groups ^58^.

SHH-MB also displays expression of coagulation and angiogenesis signaling genes compared to other MB groups. Tissue factor and protease-activated receptor 1 (thrombin receptor), essential coagulation and angiogenesis factors, are upregulated in SHH-MB ^72^. It has been shown that the extracellular protease Serpine2 regulates MB progression in *Ptch1* mice by maintaining preneoplastic lesions in a proliferative state ^73^, although the mechanism is unknown. Finally, MB therapies also have an impact on tumor vasculature. For example, irradiation induces angiogenesis in a matrix metalloproteinase 9-dependent manner in MB ^74^.

An interesting avenue of research is to determine the potential cell–cell interactions in the perivascular niche and the crosstalk between vascular structures and MB stem cells. MB stem cells (Nestin ^+^, Prominin ^+^) are closely associated with capillaries in the perivascular niche, where endothelial cells from the tumor vasculature secrete factors that promote stem cell self-renewal and propagation *in vitro* and *in vivo* ^75^. The Akt/Pten signaling pathway controls self-renewal of neural stem cells (NSCs) ^76^. Additionally, NSCs of the perivascular niche are resistant to radiation therapy, a response mediated by activation of the Akt/mTOR pathway ^77^ and which might also involve YAP1 ^78^. Pten inactivation in Nestin ^+^ cells in early postnatal periods causes the expansion of perivascular cells and, in combination with *p53* inactivation, leads to Shh-MB ^79^. Together, these studies highlight a critical role for the perivascular niche in MB initiation, growth, and resistance. The facts that Wnt signaling is essential for stem cell function and that Wnt ligands are produced by endothelial cells might explain why MB stem cells reside in the perivascular niche.

### Microenvironment invasion and metastasis

Three essential steps regulate metastasis: initiation, dispersal, and colonization. MBs most often display meningeal metastases, a process called leptomeningeal dissemination ^80^. While the cerebrospinal fluid may be the most favorable route of dissemination in MB, a hematogenous route has been demonstrated for MB ^81^. Hepatocyte growth factor (HGF), a potent inducer of epithelial-to-mesenchymal transition (EMT), induces aggressive MB in collaboration with Shh. The chemokine CCL2 and its receptor CCR2 were expressed at higher levels in MB metastases compared to primary MBs. Expression of either CCL2 or CCR2 dramatically increased leptomeningeal metastasis in a model of Shh-MB ^81^. The kinase PERK has been shown to enhance MB cell migration and invasion *in vitro* in a VEGF signaling-dependent manner ^82^. These two studies show that regulators of the tissue microenvironment can contribute to metastasis in MB.

### Cell senescence, inflammation, and stem cells

In other cancers or tissue contexts, an interesting link between senescence and cell reprogramming has been discovered ^83^. Transient exposure to senescence-associated secretory phenotype (SASP) factors, chemokines produced by senescent cells, can induce the expression of stem cell genes (such as *Nestin*, *Lgr6*, and *CD34* and cell plasticity in keratinocytes and pancreatic epithelial cells ^84^. This response promotes tissue regeneration. Conversely, *in vivo* expression of reprogramming factors induces both senescence and reprogramming; tissue damage and the ability to undergo senescence foster the reprogramming potential ^85^. Moreover, cells induced to senesce exhibit plasticity and a greater tumor-initiating potential when they escape senescence than cells that were never senescent ^86^. These results raise the possibility that senescent and inflammatory cells found within MBs could impact MB tumor-initiating cells (stem cells). In this context, increased Wnt signaling in senescent cells due to epigenetic changes is the main regulator of stemness ^86^. Given that the Wnt pathway is a major physiological regulator of stem cell self-renewal during development in many different tissue contexts such as intestinal crypts, hematopoietic stem cells, hair follicle buds, and neuroepithelial cells ^87, 88^, this suggests that senescent cancer cells might co-opt the Wnt pathway to reactivate stemness and evade senescence ^83^. This phenomenon of Wnt re-activation has been documented in cases where progenitor cells (not stem cells) become cancer cells upon oncogene activation ^88^.

Several stem cell/progenitor populations have been described in SHH-MB. Besides the EGL containing GCPs, the cerebellum displays a ventricular zone containing NSCs that give origin to all cerebellar cell types other than glutamatergic neurons ^89^. The classical marker expressed by NSCs is the intermediate filament Nestin. Additionally, NSCs are positive for GFAP and subsets of them seem to express Sox2 and Olig2, and constitutive Hh activation in this population causes SHH-MB that first transit through a GCP cell fate ^33, 90^. The early postnatal cerebellum also contains a population of Prominin (CD133)-positive stem cells ^91^ located close to the white matter that seem to be Nestin ^+^. Deletion of *p53* and *Retinoblastoma* in these Prominin ^+^ stem cells leads to MB ^92^, although with low efficiency. Moreover, a subset of Atoh1 ^+^ and CD133 ^–^ GCPs express high levels of the glycoprotein CD15 and have been shown to be tumor-propagating cells in *Ptch1 ^+/–^* MB ^93, 94^. Together, these results indicate that ventricular zone NSCs and GCPs from the EGL are the main cells with the potential to give origin to SHH-MB. Additionally, a subpopulation of Nestin ^+^ GCPs located in the inner layer of the EGL has been shown to display increased tumorigenic potential compared to Atoh1 ^+^ GCPs ^95^; these cells are Shh responsive, and it was later proposed that Nestin has the ability to bind Gli3 and prevent its conversion to a repressive form, leading to higher Hh pathway activity ^96^.

Furthermore, a population of SHH-MB tumor-propagating cells expressing Sox2 ^+^ was described as the source of MB repopulating cells subsequent to therapy ^97^. While Sox2 ^+^ cells were insensitive to Hh pathway inhibition, they displayed Wnt pathway activation, and modulation of Wnt signaling regulated their tumor-propagating abilities ^98^. In this way, Sox2 ^+^ MB cancer cells are similar to other cancer stem cells and NSCs of the cerebellar ventricular zone, which proliferate in response to Wnt signaling ^99^. Although Sox2 ^+^ MB cells are tumor-propagating cells present in primary Shh-MBs, their origin and whether they are derived from ventricular NSCs are unknown. Because Bergmann glia express Sox2, this cell type is a potential source of Sox2 ^+^ MB cancer-propagating cells.

Although insensitive to Hh pathway inhibition, Sox2 ^+^ cells seem to be specific to SHH-MB; moreover, activation of Hh signaling genes (such as *SmoM2* mutation) increased the number of Sox2 ^+^ cells in the cerebellum ^90^, suggesting that some Hh-dependent process underlies the generation of Sox2 ^+^ MB cells. The polycomb protein Bmi1 is a key regulator of stem cell function. Hh signaling activation induces Bmi1 expression ^100^ and Bmi1 is required for normal GCP expansion ^101^. While Bmi1 overexpression is insufficient to cause MB ^102^, its deletion completely prevents the formation of MB in the SmoA1 model ^103^; this effect was due to impaired progression of tumor lesions (preneoplasia), which displayed increased numbers of apoptotic and p19 ^Arf+^ cells and reduced proliferation. Whether Bmi1 is expressed or plays a role in Sox2 MB cells is unknown, but it is interesting to speculate that Hh signaling-dependent regulation of Bmi1 and other potential stem cell regulators could be the source of MB Sox2 ^+^ cells.

## Conclusion

Because of the histological similarities between tumors and stromal components undergoing tissue remodeling, pathologists since the times of Virchow have traditionally described tumors as “wounds that do not heal” ^104^; the recent recognition of the multiple roles of the tumor microenvironment in cancer confirms this idea and enables new potential ways of understanding tumors. Relative to other cancers, little is known about how the tumor microenvironment regulates MB formation. However, the identification of critical steps regulating tumor formation, such as progression, offers an interesting framework to address how the recently identified tumor cell components regulate MB formation.

Among these microenvironment cell components, clear roles are currently being established for some cell types. Macrophages seem to have a tumor-suppressive role in Shh-MB, although the specific function of macrophage subtypes, and the relative contribution of microglia, needs better understanding. Less is known about the roles of lymphocytes, although initial studies suggest that MB cells inhibit T-cell function. Besides immune cells, glia play an important role in MB formation in which astrocytes secrete Shh, promoting MB cell proliferation.

Tumor and stromal cells are regulated by signaling molecules in different ways. Shh promotes the formation and maintains the integrity of the BBB, therefore influencing factors like vascular permeability and immune cell infiltration. Wnt signaling has also been shown to regulate the formation of the BBB. The interplay between Hh and Wnt signaling in the perivascular niche, and the potential ways it regulates perivascular tumor-propagating cells, is an exciting topic of research. Finally, important research has been carried out showing cell populations other than GCPs with tumor-initiating or tumor-propagating abilities. How these cell populations arise within the tumor, whether they are sensitive to inflammatory signals in the microenvironment, and how they respond to Hh and Wnt signaling are current topics of MB research.
